# Early versus late proning in non-intubated COVID-19 pneumonia

**DOI:** 10.1186/s13054-021-03838-5

**Published:** 2021-12-10

**Authors:** Vijo Poulose

**Affiliations:** Respiratory and Critical Care Medicine, Changi General Hospital, 2, Simei Street 3, Singapore, 529890 Singapore

In a recent issue of Critical Care, Kaur et al. published an interesting study comparing the outcomes of early (EP) vs late proning on awake, non-intubated COVID-19 patients with hypoxemic respiratory failure [1]. The study is a post hoc analysis of a meta-trial on awake proning in COVID-19 pneumonia, which was published in August 2021 issue of the Lancet [2]. This “meta trial” (a novel trial design) of 6 randomised, controlled trials involved 6 nations and 1126 patients and showed that proning (versus standard care) reduced the need for intubation, but had no effect on mortality.

The primary outcomes in the Kaur study were 28-day mortality and intubation rates. The results showed that EP had a substantial mortality benefit (26% vs 45%), but with no difference in intubation rates.

Now this raises a question which the authors did not elaborate on. If EP is so effective in reducing mortality, why did it not lower intubation rates? The primary benefit of proning (as compared to supine position) is achieving better oxygenation via a variety of proposed mechanisms (better pleural pressure gradients, less weight of the heart and abdominal contents, more uniform perfusion). If EP helps in the initial exudative phase of ARDS (as the authors theorize), why did it not provide an intubation benefit?

One has to assume that all or most of the cases in the EP arm that needed intubation had worsening respiratory failure. Can this slightly confusing message be attributed to the inherent weaknesses of a post hoc analysis and the fact that being a small sample size (125 patients), the trial was probably underpowered?

I look forward to hearing from the authors for further clarification.

## Data Availability

Not applicable (NA).
