# Supplementary material for: Early versus late proning in non-intubated COVID-19 pneumonia
Source: Crit Care. 2021 Dec 10;25:422. doi: 10.1186/s13054-021-03838-5 (PMC8662665; doi:10.1186/s13054-021-03838-5)
Supplement: Related texts

# Authors’ reply to “Early versus late proning in non-intubated COVID-19 pneumonia”

**Authors:** Ramandeep Kaur, J. Brady Scott, David L. Vines, Jie Li

**Affiliations:** grid.240684.c 0000 0001 0705 3621 Department of Cardiopulmonary Sciences, Division of Respiratory Care, Rush University Medical Center, 600 S Paulina St, Suite 765, Chicago, IL USA

We thank Dr. Poulose for his interest in our work. We agree with Dr. Poulose that early use of awake prone positioning should have led to an improvement in the intubation rate. However, as we reported in our study, a higher number of patients in the late awake group (18.2% vs 7.6%) died without being intubated [1]. We believe this could have been one of the contributing factors to there being no difference in the intubation rate. We agree with Dr. Poulose that the sample size was not sufficient to detect significant differences of intubation between the early versus late prone positioning group. Future randomized controlled trials are warranted and would address the limitations of our post hoc analysis. Additionally, as this randomized controlled trial [2] was conducted in the height of the pandemic, there were wide variations in intubation practices [3, 4]. Due to concerns for aerosol transmission with the use of high flow nasal cannula therapy and non-invasive ventilation, an early intubation strategy was commonly utilized during the initial phase of the study trial. Early intubation may have impacted our ability to truly detect a difference in intubation rate based on the early initiation of awake prone positioning.
